# Neuroprotective effects of DAHP and Triptolide in focal cerebral ischemia via apoptosis inhibition and PI3K/Akt/mTOR pathway activation

**DOI:** 10.3389/fnana.2015.00048

**Published:** 2015-04-22

**Authors:** Weiyun Li, Yang Yang, Zhiying Hu, Shucai Ling, Marong Fang

**Affiliations:** ^1^Institute of Neuroscience, Zhejiang University School of Medicine, HangzhouChina; ^2^Department of Obstetrics and Gynecology, Hangzhou Red Cross Hospital, HangzhouChina

**Keywords:** cerebral ischemia, DAHP, Triptolide, PI3K/Akt/mTOR, anti-apoptosis, neuroprotection

## Abstract

Triptolide (TP), one of the major active components of the traditional Chinese herb Tripterygium wilfordii Hook F, and 2, 4-diamino-6-hydroxypyrimidine (DAHP), an inhibitor of tetrahydrobiopterin (BH4) synthesis, have been reported to have potent anti-inflammatory and immunosuppressive properties. However, the protective effects of TP and DAHP on cerebral ischemia have not been reported yet. In this study, we investigated the neuroprotective effects of TP and DAHP in a middle cerebral artery occlusion (MCAO) rat model. Furthermore, we examined whether the neuroprotective effects of TP and DAHP were associated with the inhibition of apoptosis through suppressing BH4 and inducible NOS (iNOS) synthesis or the activation of the phosphoinositide-3-kinase/serine-threonine kinase Akt/mammalian target of rapamycin (PI3K/Akt/mTOR) pathway. Our results showed that pretreatments with TP (0.2 mg/kg) and DAHP (0.5 g/kg) significantly reduced ischemic lesion volume, water content, and neuronal cell death compared with the vehicle MCAO rats. In addition, compared with the MCAO group, TP, and DAHP pretreatment groups significantly reduced astrocyte numbers, caspase-3, cleaved caspase-3, and NF-κB up-regulation, while increased Bcl-2 expression. Moreover, protein expressions of PI3K, Akt, and mTOR increased, while extracellular signal-regulated protein kinases 1 and 2 (ERK1 and ERK2) phosphorylation decreased in both the TP-treated rats and DAHP-treated rats. These results demonstrate that TP and DAHP can decrease cell apoptosis in focal cerebral ischemia rat brains and that the mechanism may be related to the activation of the PI3K/Akt/mTOR pathway and inactivation of the ERK1/2 pathway. Thus our hypothesis was reached PI3K/Akt/mTOR and ERK1/2 pathways may provide distinct cellular targets for a new generation of therapeutic agents for the treatment of stroke, and TP and DAHP may be potential neuroprotective agents for cerebral ischemia/reperfusion injury.

## Introduction

Stroke is a disease of serious consequence to human health with high prevalence worldwide. The increasing number of patients who suffer from stroke is estimated to reach more than 200 million a year with almost 150 million deaths annually (Tang et al., 2012). Cerebral ischemia accounts for 87% of all stroke patients and is one of the major causes of death and the third cause of disability (Suethanapornkul et al., 2008; Urban et al., 2010; Writing Group et al., 2010). Thus, it is essential to study the mechanisms underlying cerebral ischemia, and finding effective therapeutic strategies has become an urgent task to prevent neural damage in ischemic brain injury.

Apoptosis after cerebral ischemia is one of the major pathways that lead to the process of cell death (Mattson et al., 2001). In response to oxidative stress in mitochondria, the outer membrane of mitochondria becomes permeable. This phenomenon is directly controlled by Bcl-2 family, resulting in the translocation of Bax from the cytosol to the mitochondria and the release of cytochrome c (Kuwana et al., 2002). Cytochrome c is then released from the mitochondrial inter-membrane space to the inner cytosol, leading to the formation of the apoptosome together with Apaf-1 and caspase-9. The apoptosome permits the auto-activation of procaspase-9, which is followed by the activation of procaspase-3. Active caspase-3 which is cleaved caspase-3 leads to cell apoptosis by proteolysis such as DNA fragmentation. Previous studies have demonstrated that DNA fragmentation contributes to the development of ischemic infarction (Yao et al., 2012). Thus, the ideal preventive or therapeutic approach would indeed target apoptosis after cerebral ischemia.

Previous studies have shown that activation of the phosphoinositide-3-kinase/serine-threonine kinase Akt/mammalian target of rapamycin (PI3K/Akt/mTOR) pathway may play an important role in the effects of cell proliferation and apoptosis in the brain (Annovazzi et al., 2009). The neuroprotective role of the PI3K/Akt pathway in cerebral ischemia has been widely studied (Shibata et al., 2002; Xu et al., 2008). Activation of the PI3K/Akt pathway has been proved to negatively modulate genes that promote inflammation, thrombogenicity, and vascular permeability, and thereby protect vascular function (Schabbauer et al., 2004). Activated Akt can rapidly modulate some molecular functions including mTOR. As a multifunctional collection point, mTOR regulates cytotrophy, energy supply, and signal transduction, and also promotes protein synthesis, angiogenesis and the process of the cell cycle. Given the neuroprotective role of the PI3K/Akt pathway, we hypothesized that the PI3K/Akt/mTOR pathway may be inactivated after cerebral ischemia injury. In this study we prove that this is indeed the case in the MCAO model rats.

It is widely known that protein kinase-mediated signaling cascades play a vital role in perceiving extracellular signals and evoking downstream cellular response in neural cells (Irving et al., 2000). The ERK pathway is one of such important signal systems (Li et al., 2003; Huang et al., 2010). Extracellular signal-regulated protein kinases 1 and 2 (ERK1/2), which are members of the mitogen-activated protein kinase family, have been well characterized and are known to be involved in cell survival. However, some evidences have suggested that the activation of ERK1/2 also contributes to cell death in some cell types and organs under certain conditions (Dong et al., 2004; Kim et al., 2005; Zhuang and Schnellmann, 2006). Several studies have proved that cooperative activation of ERK and Akt promote cell survival through, respectively, suppressing distinct apoptotic mechanisms, whereas other evidences have suggested that ERK also contribute to cell death through suppressing Akt, one of the anti-apoptotic signaling molecules (Xue et al., 2000; Sodhi et al., 2001; Sinha et al., 2004). Therefore, the functional relationship between ERK and Akt after ischemic insults and whether mTOR is the downstream target of Akt that regulates neuron survival in ischemic brain injury require further investigation. In this present study, we investigated spatiotemporal expressions of ERK1/2 and PI3K/Akt/mTOR signals in the rat brain after MCAO.

Tetrahydrobiopterin (BH4) is an essential cofactor for NOS activity which is derived from peroxynitrite (ONOO) during ischemia/reperfusion and contributes to ischemic brain injury. DAHP an inhibitor of BH4 synthesis, significantly decreased gastric nitric oxid (NO) release and nitrergic relaxation. Reduced infarct volume in a MCAO rat model treated with DAHP has been reported (Kidd et al., 2005; Gangula et al., 2010). Although previous studies established a deleterious role of iNOS in ischemic stroke, the regulatory mechanisms for iNOS and the biological mechanism of DAHP in MCAO remain unknown.

As a component of traditional Chinese medicine, Triptolide (TP) has been widely used in a variety of inflammatory and autoimmune disease treatments such as rheumatoid arthritis treatment for its functions in antitumor, anti-inflammatory, and immune suppression. Furthermore, the neuroprotective effect of TP on dopaminergic neurons in MPP^+^-induced hemiparkinsonian rats has also been reported (Gao et al., 2008). Also, TP restrains NF-κB signal transduction pathway in astrocyte through inhibiting the activation of microglia (Jiang et al., 2001; Zhao et al., 2005). However, the specific antagonist effects of TP on cerebral ischemia injury need further exploration.

The aim of this study was to investigate whether brief administrations of DAHP (0.2 mg/kg) and TP (0.5 g/kg) 12 h before MCAO salvage neurons from inevitable injury during reperfusion (90 min). We further tested the hypothesis that these beneficial actions are mediated activation of the PI3K/Akt/mTOR signaling pathway through inhibiting apoptotic mechanism.

## Materials and Methods

### Animals

Adult male Sprague–Dawley rats (the Animal Center of Zhejiang University, China), weighing 230–250 g, were used in the experiments. Animals were maintained under special pathogen-free conditions and have free access to sterilized water and pellet food. These rats were kept under standardized conditions with a 12 h light–dark cycle. Temperature (24 ± 1°C) and humidity (55%) remained constant. All experiments were conducted in compliance with the National Institute of Health’s Guidelines for the Care and Use of Laboratory Animals.

### Middle Cerebral Artery Occlusion

According to previous research, male Sprague–Dawley rats were subjected to perform MCAO and reperfusion operations (Longa et al., 1989). Briefly, rats were anesthetized with 4% chloral hydrate intraperitoneally. Then MCAO was carried out using an intraluminal thread introduced via common carotid artery (CCA). A surgical midline incision was made to expose the right CCA, external carotid artery (ECA), and internal carotid artery (ICA). A 4-0-monofilament nylon suture with approximately 0.26 mm round in diameter was inserted into the right CCA lumen and gently advanced into the ICA up to a point approximately 18 mm. After 90 min, the nylon sutures were slowly removed from the artery, and the animals were allowed reperfusion. Then we closed the incision on the neck. Using an automatic homeothermic blanket control unit, the animal’s body temperature was continually monitored and maintained at 37°C throughout the surgical procedure and during post-surgery recovery.

### Drug Administration and Experimental Groups

A total of 60 adult male Sprague–Dawley rats were randomly seperated into 5 groups: (1) control group (*n* = 12), (2) DMSO group (*n* = 12), (3) MCAO group (*n* = 12), (4) DAHP-treated group (*n* = 12), and (5) TP-treated group (*n* = 12). The control group (sham operated animals) underwent the MCAO surgical procedure, except the thread was not inserted into the CCA. The animals in the DMSO group were almost same with the control group except giving intraperitoneal injection of 0.5 ml DMSO. In TP-treated and DAHP-treated groups, animals went through the MCAO surgical procedure, and TP and DAHP were administrated by intraperitoneal injection (i.p.) 12 h before the beginning of MCAO at a dose of 0.2 mg/kg and 0.5 g/kg, respectively, (Kidd et al., 2005; Klawitter et al., 2012; Lee et al., 2012; Yang et al., 2015). TP and DAHP were obtained from Merck and dissolved in DMSO. All the tissue samples were collected at the same time point, 24 h after MCAO surgical procedure.

### Evaluation of Cerebral Edema and Infarction TTC

The rats were injected intraperitoneally with sodium pentobarbital (40 mg/kg). After being anesthetized, the brains were removed rapidly and frozen in -20°C for 30 min. Brains were dissected, and coronal slices (2 mm in thickness) were acquired from frozen forebrains using a rodent brain matrix slicer. Brain slices were then stained with TTC (2%) at 37°C for 20 min in dark. The sections were soaked in 4% paraformaldehyde phosphate buffer for 1 h and scanned. The percent of infarct area of the entire brain represented the degree of cerebral infarction. Normal brain tissues were stained red, while the unstained (white)area was considered to be the infract area. Areas of red and white staining were measured using a computer color multimedia image analysis system (Image J 1.46R, NIH, USA). The percent of infarction is revealed by the equation: % infarct area = infarct area/total area of slice × 100 (Cheng et al., 2012).

### MRI Examination

The rats in each group were examined by MRI to investigate whether the rats displayed signs of hemispheric swelling and oedema formation. Rat brains were then tested in a 3.0-Tesla (T) MRI animal scanner (Magnetom Trio with TIM system, Siemens, Erlangen, Germany). In order to obtain the signal excitation and detection, the rat’s head was placed in a custom-made “birdcage coil,” which has a 30 mm inner diameter. MRI parameters were set as follows: TE = 92 ms, TR = 3620 ms, FOV = 8 cm × 8 cm, *M* = 256 × 256, NA = 2, thickness = 2 mm, and gap = 0 mm. The rat was held in a flat skull position, and the brain was performed to center the image slice 5 mm posterior to the rhinal fissure. Image-Pro Plus 5.0 software (Media Cybernetic, Bethesda, MD, USA) was used to examine the hemisphere intensity by “mean density value” after the optimal adjustment of contrast. MRI measurements were obtained 24 h after MCAO.

### Immunofluorescence

Brains were perfusion fixed with 4% paraformaldehyde and 30% sucrose solution before processing for histology. After being frozen, brains were sectioned coronally. Primary antibodies were applied in the following concentrations: GFAP and iNOS (1:100; Santa Cruz Biotechnology, Inc., USA). Immunohistochemistry followed the method with HRP-conjugated goat anti-rabbit IgG (1:200; Santa Cruz Biotechnology, Santa Cruz, CA, USA), and then were visualized with 3, 30-diaminobenzidine (DAB, Sigma, St. Louis, MO, USA). Sections were then hydrated, cleared and mounted in DXP for microscopic analysis. Immunofluorescence followed with appropriate secondary antibodies (Alexa Fluor, Molecular Probes Inc.) in 1% BSA and 0.3% Triton X-100 in PBS after primary antibodies. Mounting medium was added on the slides prior to be covered with coverslips for observation by a laser scanning confocal microscope.

### Western Blot

Total proteins of the ischemic penumbra brain tissue were extracted from tissues of each group with 2 mM phenylmethanesulfonyl fluoride in 1 mL ice-cold RIPA buffer added protease inhibitor cocktail EDTA-free and phosphates inhibitors. BCA kit (KeyGEN, Nanjing, China) was used to determine protein concentration. A total of 30 μg of total protein from each sample was subjected to electrophoresis on 12% SDSPAGE gel using a constant voltage. After performing electrophoresis 70 V for 30 min and 100 V for 120 min, the proteins were electrophoretically transferred to PVDF membrane using a Bio-Rad TransBlot apparatus for 120 min. The PVDF membranes were blocked with TBST containing 5% non-fat milk for 2 h at room temperature, and then incubated with rabbit anti-ERK1/2 and Bcl-2 (1:1000; Santa Cruz Biotechnology, Santa Cruz, CA, USA), caspase-3, cleaved caspase-3, NF-κB, pERK1/2, Phospho-Akt (Ser473), and Akt (1:1000; Cell Signaling Technology, Inc., USA), mTOR and PI3K (1:1000; Epitomics, USA) primary antibody overnight at 4°C. The membrane was washed with TBS containing 0.05% Tween 20 (TBST) for 15 min and three times, then incubated with horseradish peroxidase-conjugated goat anti-rabbit (1:3000; Jackson Immuno Research Laboratories, USA) antibody for 2 h, and then washed with TBST for 15 min and three times. Finally, membranes were processed for detection using the ECL system. The band density was analyzed by Quantity One, and all experiments performed in triplicate to represent the mean plus or minus SD of all data.

### TUNEL Staining

Terminal deoxynucleotidyl transferase-mediated dUTP-biotin nick end labeling staining was assessed by the *In Situ* Cell Death Detection Kit, POD (Roche Applied Science, Mannheim, Germany). After 24 h reperfusion, the sections were prepared and the staining was performed according to the protocol provided by the manufacturer. Confocal laser scanning microscope was employed to analyze the results. The number of TUNEL positive neurons and total neurons was counted in three different fields for each section and calculated in 10 selected sections by an examiner blinded to the group assignment. The extent of apoptosis was calculated and expressed as a ratio of TUNEL-positive neurons versus total neurons (Wang et al., 2012).

### Statistical Analysis

The data were analyzed using Graph Pad Prism version 4.0. Values are presented as means ± SD for the indicated analyses. A probability value of *P* < 0.05 was considered to be statistically significant for all statistical tests. To compare mean values in two separate groups for infarct volume, we used an unpaired *t*-test. Values of *P* < 0.05 were considered significant. Western-blot had been evaluated by Quantity one analysis. Differences at *P* < 0.05 were considered statistically significant.

## Results

### DAHP and Triptolide Improve Edema

**FIGURE 1:**
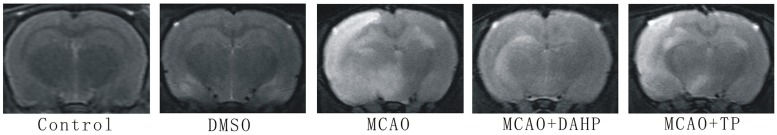
**T2-weighted MRI images of rats’ brain**.

Magnetic resonance imaging is considered to be the most promising and non-invasive approach for examining brain edema. Assessment of hemispheric volumes on MRI allows a direct quantification of the space-occupying effect in experimental stroke. T2-weighted MRI is frequently used to determine the edema induced by I/R. The representative images were shown in **Figure 1**. Brain edema was hardly observed in the animals of the control group. In contrast, edema was detected at 24 h after reperfusion in MCAO group. The brain edema was less in both the DAHP-treated and TP-treated groups.

### Triptolide and DAHP Reduce Infarction Volume and Brain Water Content

**FIGURE 2:**
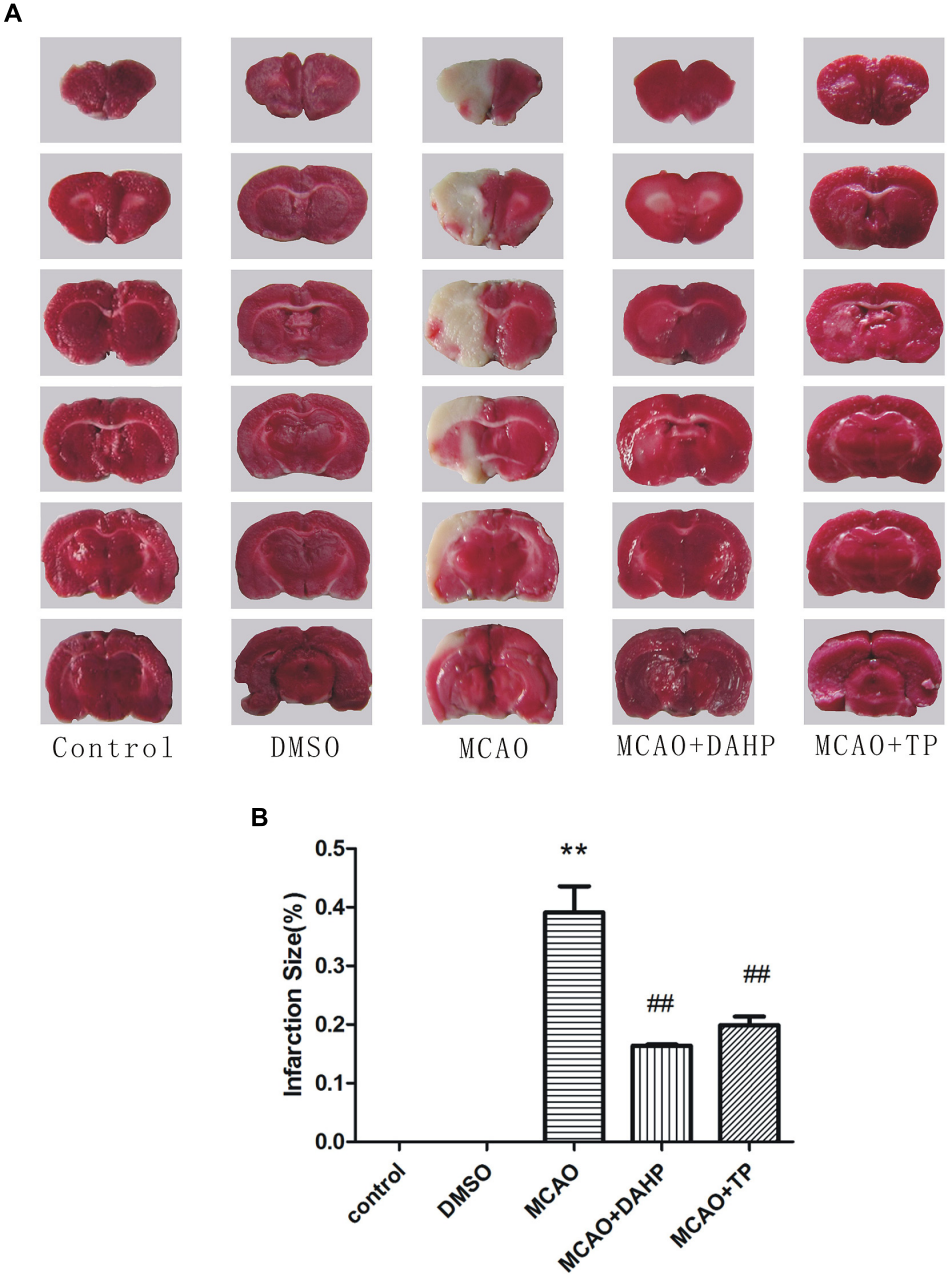
**2, 4-diamino-6-hydroxypyrimidine and Triptolide (TP) reduced focal cerebral ischemia-induced injury. **(A)** Representative photographs of coronal brain sections stained with 2,3,5-triphenyltetrazolium chloride. (B)** Summary of cerebral infarct size in brains. The infarct volume was expressed as the percentage of the contra lateral hemispheric area. Infarction size was then analyzed using Meta-Morph program. Data were expressed as mean ± SD, **P < 0.01 vs. control group; ##*P* < 0.01 vs. MCAO group.

2, 3, 7-triphenyltetrazolium chloride staining reflected the neurological deficit in the rat brain. The representative images are presented in **Figure 2A**. There were significant differences in lesion areas between rats in the control group and MCAO groups (*P* < 0.01). The lesion area in the TP and DAHP pre-treatment groups were reduced noticeably compared with the model group (*P* < 0.01). The area of the lesion in the DMSO group showed no significant difference from the model group. In the TP and DAHP treated groups, the area of the lesion was reduced, but no significant difference was found when compared with the DMSO group in **Figure 2B**.

### Triptolide and DAHP Reduces Apoptotic Cell Death in the Brain

**FIGURE 3:**
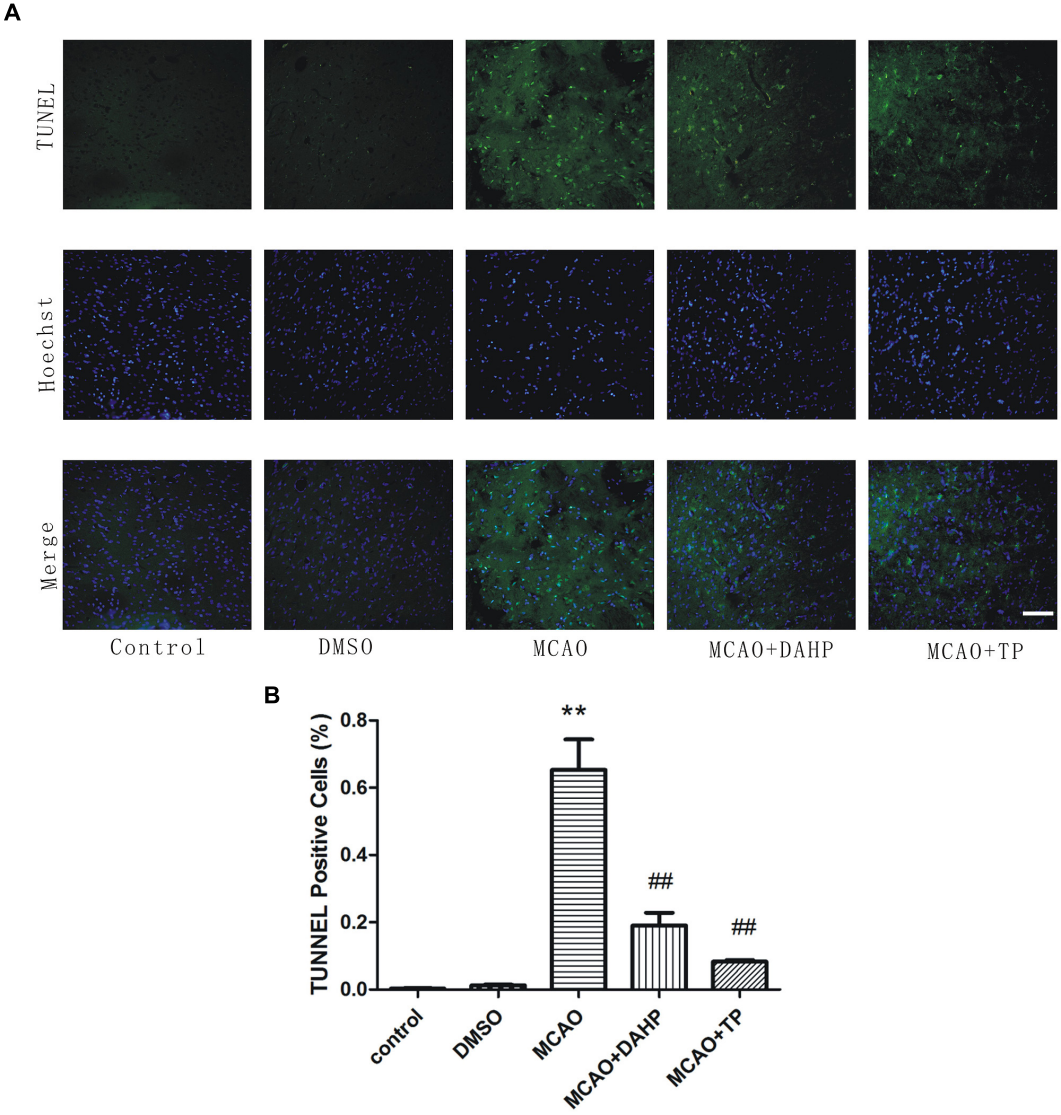
**Detection of neuronal apoptosis. (A)** TUNEL staining was used to identify apoptotic cells. **(B)** TUNEL positive cells were counted using Meta-Morph program. TUNEL positive cells show the quantitative analysis data. Data are expressed as mean ± SD, **P < 0.01 vs. control group; ##*P* < 0.01 vs. MCAO group.

At the 24 h time point after reperfusion, the number of apoptotic cells in penumbral area was observed. Apoptotic cells were recognized by TUNEL which is the identification of DNA fragmentation. As can be seen from **Figure 3**, in the MCAO and DMSO group, the numbers of TUNEL positive cells were significantly increased, as compared to sham rats. In the TP-treated and DAHP treated group, the numbers of TUNEL positive cells were significantly decreased, as compared to the MCAO group. These results suggested that the TP-treated and DAHP-treatment effectively prevented expansion of apoptotic cell death in MCAO model. Data is expressed as mean ± SD, **P < 0.01 vs. control group; ##*P* < 0.01 vs. MCAO group.

### Triptolide and DAHP Reduce Glial Activation

**FIGURE 4:**
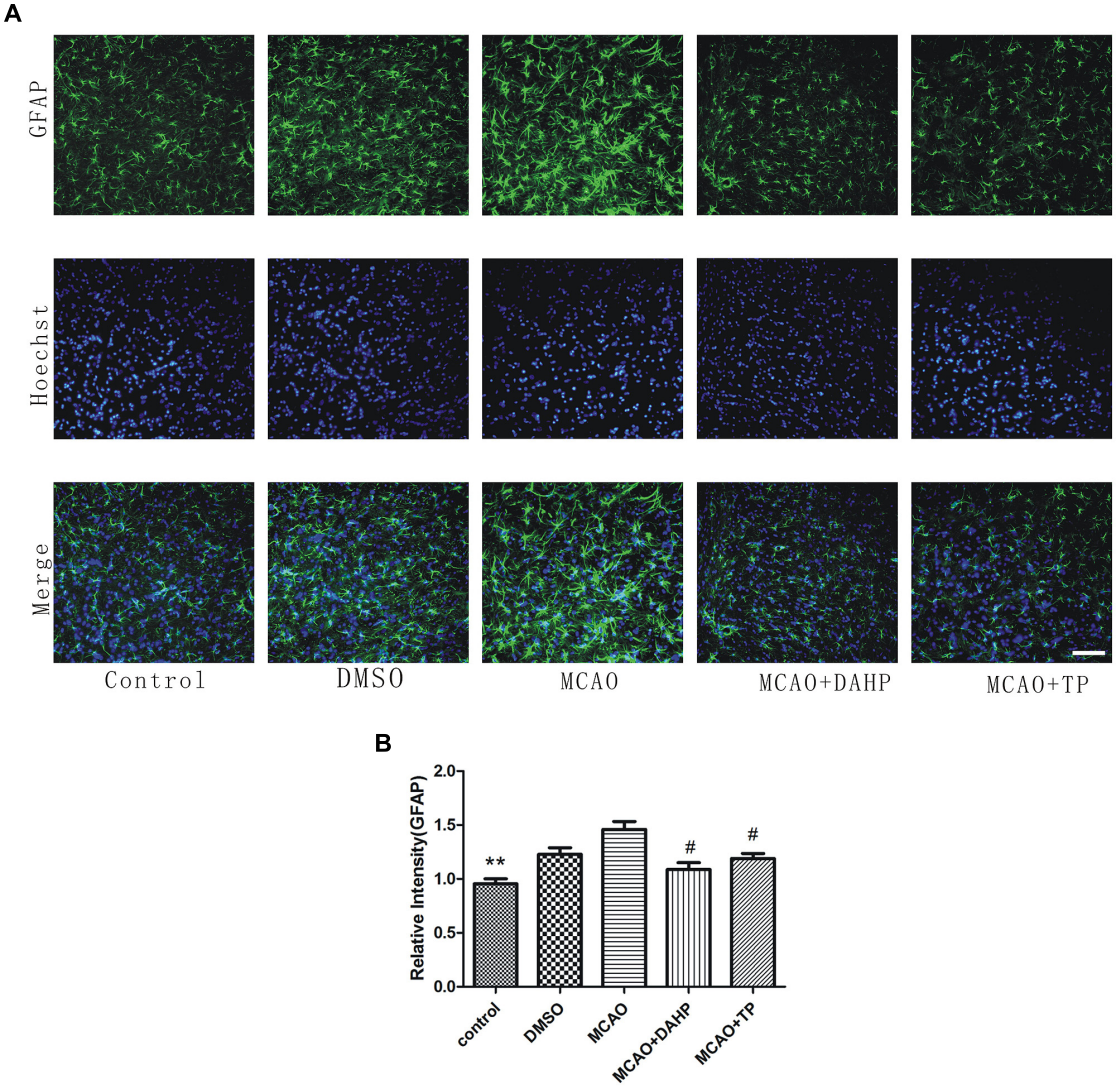
**2, 4-diamino-6-hydroxypyrimidine and TP treatments attenuated ‘reactive gliosis’ in the ischemic area. (A)** Immunofluorescence staining showed immunoreactivities of GFAP after MCAO. The profound expression of GFAP was observed in MCAO group compared to control group **(B)**. The expression of GFAP in DAHPtreated and TP-treated group was significantly decreased compared with MCAO group. **P < 0.01 vs. MCAO group; #*P* < 0.05 vs. MCAO group.

To specifically investigate the effect of MCAO on astrocyte function and TP and DAHP on the neuroinflammatory system, we measured the expression of GFAP following ischemic insult by immunofluorescence staining analysis at 24 h after reperfusion. The number of GFAP-positive astrocytes was observed. In the MCAO group, the GFAP-positive astrocytes were mainly gathered in the penumbral area. In TP-treated and DAHP-treated rats, the number of GFAP-positive astrocyte accumulation significantly decreased compared to the MCAO rats (**Figure 4**). The result showed that the inhibitory effects of TP and DAHP on GFAP-positive astrocyte might contribute to its neuroprotective effect in MCAO. **P < 0.01 vs. MCAO group; #*P* < 0.05 vs. MCAO group.

### Triptolide and DAHP Attenuate the Increasing of iNOS

**FIGURE 5:**
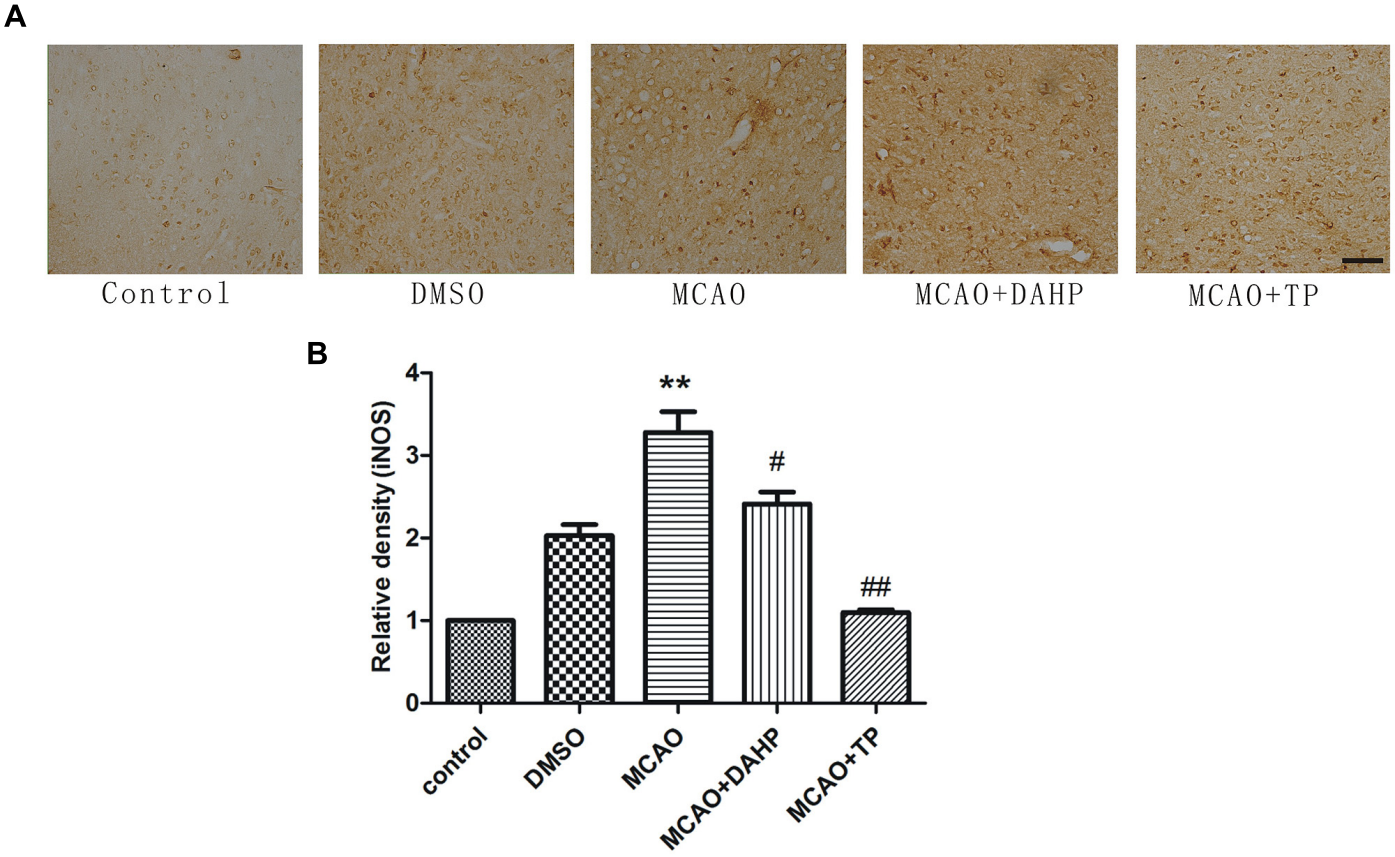
**2, 4-diamino-6-hydroxypyrimidine and TP treatments attenuated iNOS expression in ischemic area. (A)** Immunohistochemistry showed immunoreactivities of iNOS after MCAO. The profound expression of iNOS was observed in MCAO group compared to control group **(B)**. The expression of iNOS in DAHP-treated and TP-treated group was significantly decreased compared with MCAO group. **P < 0.01 vs. control group; ##*P* < 0.01 vs. MCAO group; #*P* < 0.05 vs. MCAO group.

As shown in **Figure 5A**, immunochemistry results revealed that stroke increased the iNOS activity when compared with non-ischemic rats. Ischemic reperfusion induced a substantial increase in iNOS activity, which was significantly attenuated by TP and DAHP. In comparison with DAHP, the extent of reduction of increased iNOS activity induced by ischemic reperfusion was higher by TP treatment (**Figure 5B**). Immunoreactive stainings of iNOS was detected in the cerebral cortex. The staining for iNOS was moderately expressed in the ischemic penumbral cortex (**Figure 5A**). **P < 0.01 vs. control group; ##*P* < 0.01 vs. MCAO group; #*P* < 0.05 vs. MCAO group.

### Triptolide and DAHP Activated the PI3K/Akt/mTOR Pathway and Increased the Expression of Bcl-2

**FIGURE 6:**
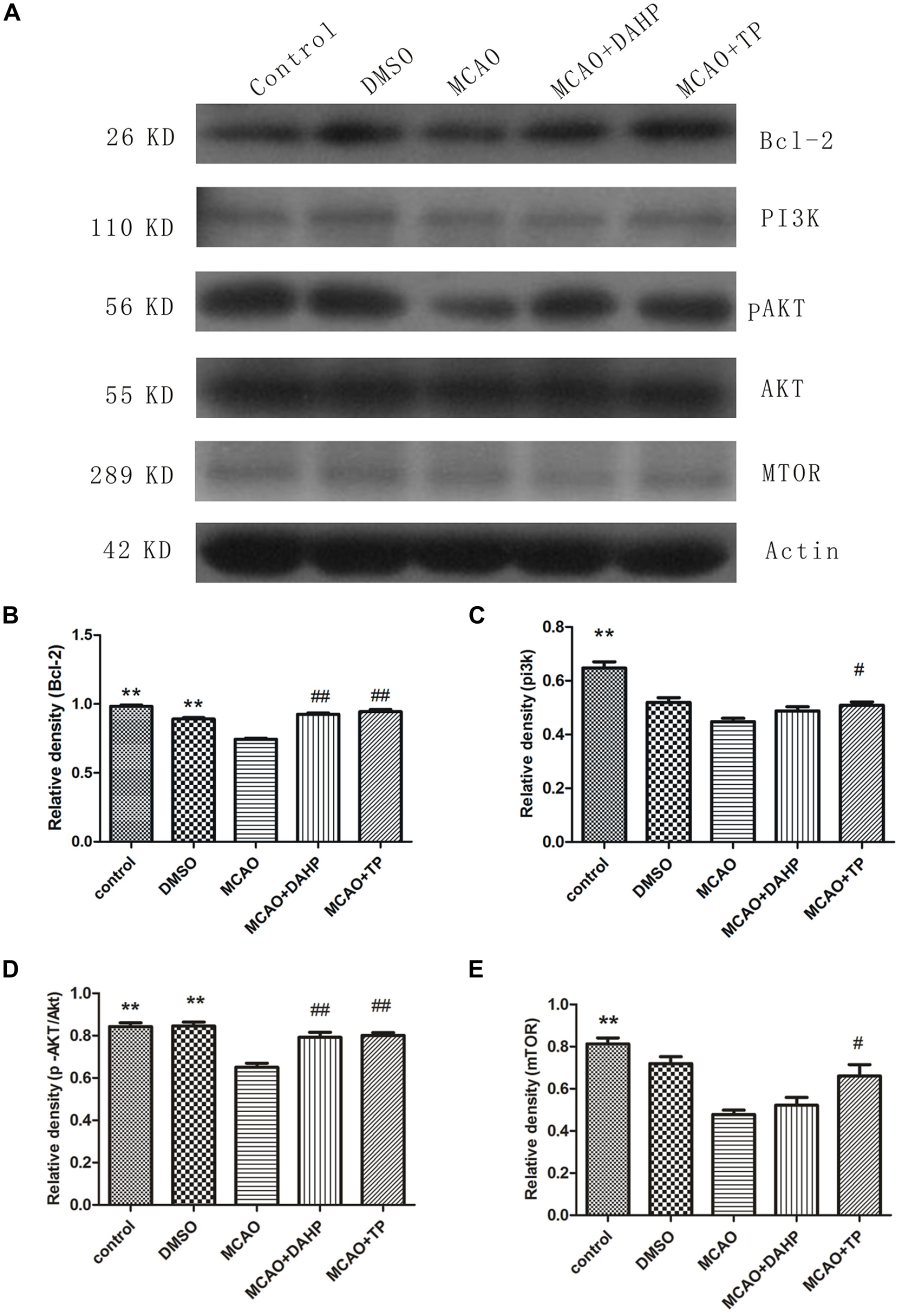
**2, 4-diamino-6-hydroxypyrimidine and TP treatments rescued the decreased levels of PI3K, phosphorylated Akt Ser-473, and mTOR induced by ischemia in the cortex and hippocampus, also restored the decreased level of Bcl-2 induced by ischemia. (A)** Compared with the control group, the PI3K, Akt, and mTOR protein levels decreased in the MCAO group. And it restored the decreased level of Bcl-2 **(B)** induced by ischemia DAHP and TP up-regulated the protein levels of PI3K **(C)**, Akt **(D)**, and mTOR **(E)**. **P < 0.01 vs. MCAO group; ##*P* < 0.01 vs. MCAO group; #*P* < 0.05 vs. MCAO group.

Western blot analysis revealed that stroke decreased the activity of PI3K/Akt/mTOR pathways compared with non-ischemic rats. Treatments with TP and DAHP significantly increased PI3K, Akt and mTOR expression compared to the MCAO group (**Figures 6A,C–E**). Compared with the sham-operated rats, the protein expression of Bcl-2 significantly decreased in the MCAO rats (**Figure 6B**). Pretreatments of TP and DAHP significantly increased the protein expression of Bcl-2 TP and DAHP vs. MCAO group. **P < 0.01 vs. MCAO group; ##*P* < 0.01 vs. MCAO group; #*P* < 0.05 vs. MCAO group.

### Triptolide and DAHP Decreased the Expression of Caspase-3, NF-κB, and Cleaved Caspase-3

**FIGURE 7:**
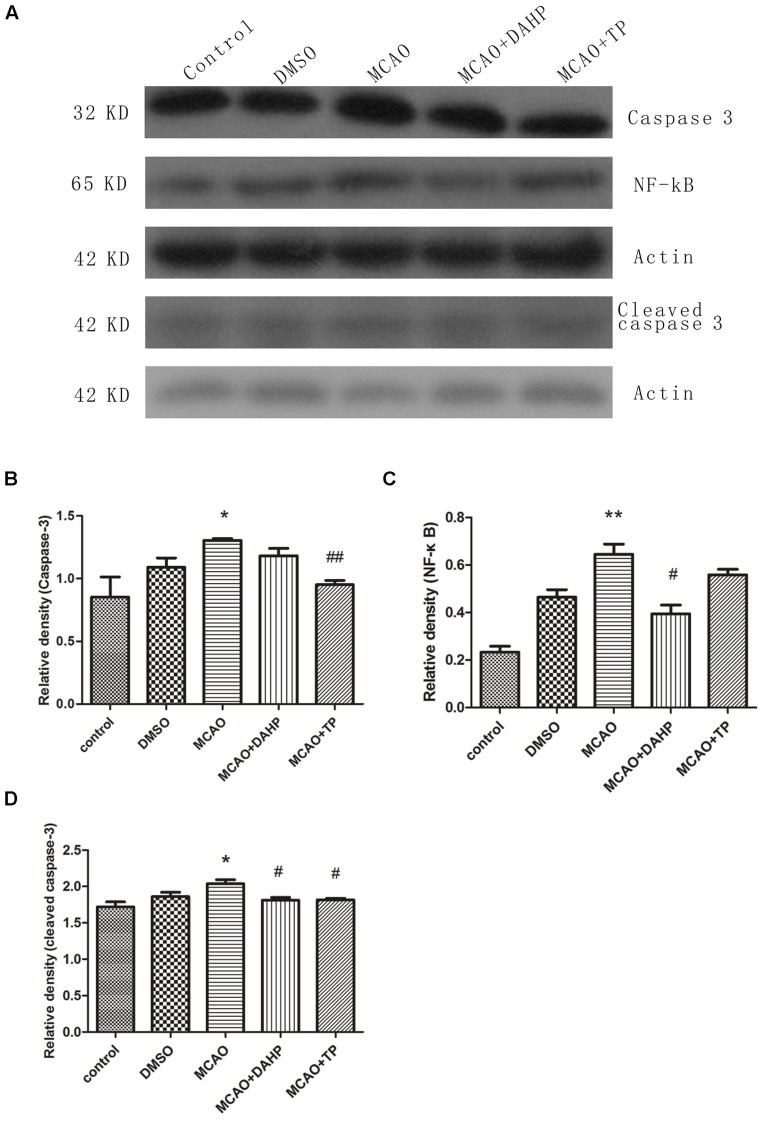
**2, 4-diamino-6-hydroxypyrimidine and TP treatment inhibited the expressions of caspase-3 **(A)**, NF- κB and cleaved caspase-3**. The expression of caspase-3 **(B)**, NF-κB **(C)** and cleaved caspase-3 **(D)** was increased in MCAO group compared to control group. DAHP and TP treatment significantly reduced the expression of caspase-3, NF-κB and cleaved caspase-3. **P < 0.01 vs. control group; *P < 0.05 vs. control group; #*P* < 0.05 vs. MCAO group; ##*P* < 0.01 vs. MCAO group.

**Figure 7A** shows the results of western blot with caspase-3, NF-κB and cleaved caspase-3 antibodies in cortex of rats 24 h after MCAO. Increased protein level of NF-κB was observed 24 h after MCAO. TP and DAHP decreased the protein expressions of NF-κB. Also, the expressions of caspase-3 and cleaved caspase-3 were analyzed by western blot. The data showed that TP and DAHP significantly decreased the protein expressions of caspase-3 and cleaved caspase-3 (**Figures 7A–D**). **P < 0.01 vs. control group; *P < 0.05 vs. control group; #*P* < 0.05 vs. MCAO group; ##*P* < 0.01 vs. MCAO group.

### Triptolide and DAHP Decreased pERK1/ERK1 and pERK2/ERK2 Expressions

**FIGURE 8:**
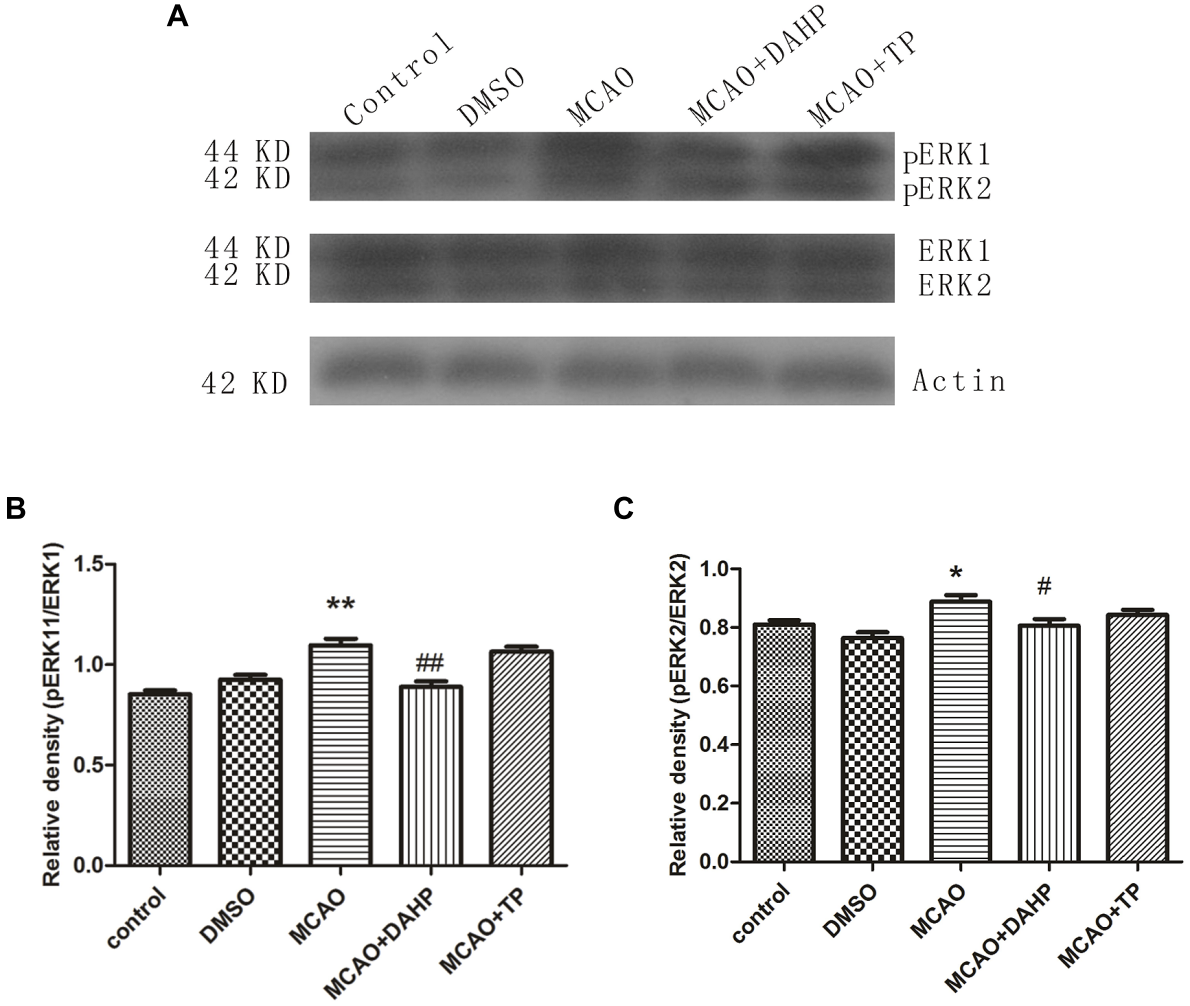
**2, 4-diamino-6-hydroxypyrimidine and TP treatment attenuated the increasing expressions of pERK1/ERK1 and pERK2/ERK2. (A)** The expressions of pERK1/ERK1 and pERK2/ERK2 were increased in MCAO group compared to control group. DAHP and TP treatment significantly attenuated the increasing expressions of pERK1/ERK1 **(B)** and pERK2/ERK2 **(C)**. **P < 0.01 vs. control group; *P < 0.05 vs. control group; #*P* < 0.05 vs. MCAO group; ##*P* < 0.01 vs. MCAO group.

ERK1, ERK2, phospho-ERK1 (pERK1), and phospho-ERK2 (pERK2) expressions were examined after MCAO using western blot and immunofluorescence staining. The results showed that ERK1, ERK2, pERK1, and pERK2 were detected after 90 min of MCAO in both the ischemic core and the perifocal regions. Western blot analysis showed that levels of pERK1/ERK1 and pERK2/ERK2 were obviously increased after 90 min of MCAO in comparison with sham-operated group. Treatments with TP and DAHP decreased the expressions of pERK1/ERK1 and pERK2/ERK2 (**Figures 8A–C**). **P < 0.01 vs. control group; *P < 0.05 vs. control group; #*P* < 0.05 vs. MCAO group; ##*P* < 0.01 vs. MCAO group.

## Discussion

The research for neuroprotective agents in MCAO is primarily based on the abilities of specific drugs to reduce neuronal loss and contribute to cell survival. Agents that can provide such neuroprotective effects have the potential to become novel therapeutics for MCAO. Existing animal models of MCAO provide us with useful tools to test the two new neuroprotective agents and strategies. In this *in vivo* ischemia study the spatiotemporal profile of PI3K/Akt/mTOR pathway was examined in a rat transient MCAO model to investigate the mutual role of signal pathways.

Cerebral ischemia significantly promoted the neurological damage and increased total infarct size. The results showed that rats undergoing ischemic stress exhibited a high percentage of TUNEL-positive cells; however, these were markedly decreased in the peri-infarct area in TP-treated and DAHP-treated rats. These observations suggest that apoptosis is one of the major mechanisms that leads to cell death in the ischemic cortex. Several studies have suggested that inhibiting glial activation attenuates ischemic injury and neuronal death (Suk, 2004; Wang et al., 2005). Like many other neurodegenerative disorders, the reactive gliosis associated with ischemic stroke involves astrocytes (Kriz, 2006). Reactive gliosis can produce excess amounts of cytokines as well as inflammatory products that exacerbate ischemic damage (Walker and Rosenberg, 2009). In the present study, TP and DAHP suppressed the increasing number of GFAP-positive cells in the peripheral area and central ischemic core of the ischemic infarct. There are papers reported that NO derived from iNOS contributes to neuronal injury after cerebral ischemia reperfusion, during which NO interacts with superoxide anion (O_2_) to form ONOO and causes neuronal death (Oliver et al., 1990). An increase in iNOS protein expression has been proved after cerebral ischemia in this paper, which is consistent with that *in vivo* DAHP can reduce cerebral infarction via inhibiting iNOS activity and ONOO level in transient focal ischemia, and iNOS inhibitor can protect against neuronal injury in ischemic stroke (Kolinsky and Gross, 2004; Kidd et al., 2005). In contrast to DAHP, TP plays a more positive role in decreasing iNOS activity. These results indicate that the neuroprotective effects of TP and DAHP may be mediated by effectively preventing against infarct expansion through secondary injury caused by activated astrocytes and iNOS-induced oxidative stress in focal cerebral ischemia.

Neural cell death resulting from cerebral ischemia involves both the necrotic and apoptotic mechanisms. Extracellular signals often result in simultaneous activation of Akt pathway, and many reports suggest a survival role of Akt signal pathway through suppression of apoptosis (Xue et al., 2000; Li et al., 2003). There have been evidenced that Akt indirectly activates NF-κB transactivation by dissociation of phosphorylated Iκ-B kinase, resulting in transcription of pro-survival genes like B-cell lymphomaextra large (Bcl-xL) and trophic factors (Jeon et al., 2012). The expression of pro-survival factors such as Bcl-2 and Akt decreased after ischemic injury but increased in TP-treated and DAHP-treated groups. This shows that apoptosis plays a vital role in stroke and also the anti-apoptosis effects of TP and DAHP.

Activation of apoptotic pathways is always concomitant with the survival signaling pathways. PERK1/ERK1, pERK2/ERK2, pAkt/Akt, and mTOR expressions suggest that ERK1/2 and PI3K/Akt/mTOR signal pathways play essentially an independent role, especially after ischemic insults. The PI3K/Akt signaling pathway plays a key role in many cellular processes, including cell survival, coagulation, and inflammation responses. Furthermore, activation of the PI3K/Akt pathway in cerebellar granule neurons has been proposed to prevent neuronal cell death by suppressing JNK activation (Shimoke et al., 1999). In the present study, we demonstrated that pAkt and mTOR significantly decreased in rats after ischemic injury, but increase markedly in TP-treated and DAHP-treated groups. MTOR promotes angiogenesis, neuronal regeneration, and synaptic plasticity. It can further reduce neuronal apoptosis, and remove neurotoxic substances, which are all closely associated with the repair and survival mechanisms of ischemic brain injury (Chen et al., 2012a). And there has been demonstrated that mTOR inhibition by rapamycin increased neuronal apoptosis in the early phase of neonatal HI brain injury, leading to an increase in cleaved caspase-3 (Chen et al., 2012b). These results thus indicate that PI3K/Akt/mTOR pathways may play an important role in the extrinsic apoptosis pathway regulating neurotoxic substances and inflammation responses in ischemic brain injury, consequently resulting in increased neuronal survival. Recently, it was reported that ERK1/2 activation that was accompanied by a gradual decrease in Akt activity and apoptosis and inhibition of ERK1/2 prevented the decline in Akt activity and resulted in cell survival (Sinha et al., 2004). These results may suggest that ERK1/2 activation in response to survival factor deprivation contributed to cell death via suppressing the PI3K/Akt/mTOR pathway. Several studies have revealed that the intrinsic pathway is characterized by mitochondrial outer membrane permeabilization, cytochrome c release, caspase-3 activation, DNA fragmentation, and death-inducible signaling complex formation, and these events have been shown to be associated with ERK activation (Wang et al., 2000; Jo et al., 2005; Kim et al., 2005). In the present study, caspase-3, cleaved caspase-3, ERK1, and ERK2 expression increased markedly under chemic stress, while decreased in groups treated with TP and DAHP. Pre-treatments of animals with TP and DAHP prior to ischemic injury contributed to the decreased ERK1/2 staining, which may prevent primary neuronal damage. Further investigations are required to clarify more details of these molecular balances with other signaling systems that lead cells to survival or death.

In summary, many signal transduction systems are regulated following the onset of focal cerebral ischemia, some of which may be protective and some may stimulate subsequent cellular degeneration. Activation of the PI3K/Akt/mTOR pathway plays an essential mechanism for maintaining cellular homeostasis when responding to focal cerebral ischemia. Activation of the ERK1/2 pathway has been implicated in the mechanisms underlying cerebral ischemia, i.e., decreased lesion volumes have been demonstrated following the administration of specific inhibitors (Irving et al., 2000; Irving and Bamford, 2002). However, data presented here clearly indicates that the ERK1/2 pathway is activated in a different spatial location within the ischemic brain. Both pre-treatments of TP and DAHP clearly demonstrated their protective effects in focal cerebral ischemia through activation of PI3K/Akt/mTOR pathway and inactivation of ERK1/2 pathway. DAHP may provide neuroprotection through inhibiting iNOS-induced oxidative stress within neurons and glia, thus directly protecting these cells. In contrast, TP may exert protection of all cellular elements via the inhibition of the brains inflammatory response to injury and immune suppression. These findings provide a mechanistic basis for new therapeutic strategies aiming to regulate PI3K/Akt/mTOR and ERK1/2 pathways in order to prevent against injuries induced by focal cerebral ischemia. And TP and DAHP may be considered to be potential therapies for focal cerebral ischemia.

## Author Contributions

MF designed the experiments and WL drafted the manuscript, YY and ZH participated in the study design and coordination, WL and YY performed the experiments, SL and ZH analyzed the data and revised the manuscript. All authors read and approved the final manuscript.

## Conflict of Interest Statement

The authors declare that the research was conducted in the absence of any commercial or financial relationships that could be construed as a potential conflict of interest.

## Abbreviations

DAHP: 2, 4-diamino-6-hydroxy-pyrimidine
DMSO: dimethyl sulfoxide
ERK: extracellular signal-regulated kinase
MCAO: middle cerebral artery occlusion
MRI: magnetic resonance imaging
mTOR: mammalian target of rapamycin
PI3K: Class III phosphatidylinositol kinase
PVDF: polyvinylidine difluoride
TTC: 2, 3, 7-triphenyltetrazolium chloride
TUNEL: terminal dexynucleotidyl transferase (TdT)-mediated dUTP nick end labeling.
